# Transcriptomic expression profiling identifies ITGBL1, an epithelial to mesenchymal transition (EMT)-associated gene, is a promising recurrence prediction biomarker in colorectal cancer

**DOI:** 10.1186/s12943-019-0945-y

**Published:** 2019-02-04

**Authors:** Takatoshi Matsuyama, Toshiaki Ishikawa, Naoki Takahashi, Yasuhide Yamada, Masamichi Yasuno, Tatsuyuki Kawano, Hiroyuki Uetake, Ajay Goel

**Affiliations:** 1 Center for Gastrointestinal Research, Center for Translational Genomics and Oncology, Baylor Scott & White Research Institute and Charles A. Sammons Cancer Center, Baylor University Medical Center, Dallas, 3410 Worth Street, Suite 610, Dallas, TX 75246 USA; 2 Department of Specialized Surgery, Tokyo Medical and Dental University Graduate School of Medicine, Tokyo, Japan; 3 Department of Gastroenterology, National Cancer Center Hospital, Tokyo, Japan; 4 Department of Gastrointestinal Surgery, Tokyo Medical and Dental University Graduate School of Medicine, Tokyo, Japan; 5 Department of Gastroenterology, Saitama Cancer Center Hospital, Saitama, Japan

**Keywords:** ITGBL1, Prognostic marker, Epithelial mesenchymal transition, Colorectal cancer

## Abstract

The current histopathological risk-stratification criteria in colorectal cancer (CRC) patients following a curative surgery remain inadequate. In this study, we undertook a systematic, genomewide, biomarker discovery approach to identify and validate key EMT-associated genes that may facilitate recurrence prediction in CRC. Genomewide RNA expression profiling results from two datasets (GSE17538; *N* = 173 and GSE41258; *N* = 307) were used for biomarker discovery. These results were independently validated in two, large, clinical cohorts (testing cohort; *N* = 201 and validation cohort; *N* = 468). We performed Gene Set Enrichment Analysis (GSEA) for understanding the function of the candidate markers, and evaluated their correlation with the mesenchymal CMS4 subtype. We identified integrin subunit beta like 1 (ITGBL1) as a promising candidate biomarker, and its high expression associated with poor overall survival (OS) in stage I-IV patients and relapse-free survival (RFS) in stage I-III patients. Subgroup validation in multiple independent patient cohorts confirmed these findings, and demonstrated that high *ITGBL1* expression correlated with shorter RFS in stage II patients. We developed a RFS prediction model which robustly predicted RFS (the area under the receiver operating curve (AUROC): 0.74; hazard ratio (HR): 2.72) in CRC patients. ITGBL1 is a promising EMT-associated biomarker for recurrence prediction in CRC patients, which may contribute to improved risk-stratification in CRC.

**Electronic supplementary material:**

The online version of this article (10.1186/s12943-019-0945-y) contains supplementary material, which is available to authorized users.

Colorectal cancer (CRC) remains one of the primary causes of cancer-related deaths worldwide [1]. Although surgery remains the best treatment choice, a significant majority of stage II and III CRC patients develop disease recurrence following a curative resection; highlighting the inadequacy of currently used TNM classification for patient prognostication. Due to the high recurrence rates, patients with stage III disease routinely receive adjuvant chemotherapy [2]. Even though a clear benefit of adjuvant treatment in stage II CRC patients remains debatable, adjuvant chemotherapy is thought to be a reasonable treatment modality for the subgroup of high-risk stage II patients [3]. Nonetheless, given the relatively poor therapeutic response and high cancer recurrence rates, the current histopathological risk-stratification criteria remain inadequate. To address this concern, researchers have attempted to develop various biomarkers for patient stratification [4]; however, due a variety of biological and technical reasons, most of these biomarkers fail independent validations and are hence still not adopted in the clinical settings.

Epithelial-to-mesenchymal transition (EMT) is considered an essential regulatory process that mediates invasion and metastasis in cancer [5]. Recently, four consensus molecular subtypes (CMS) were identified in CRC patients following a comprehensive gene expression profiling [6]. Among these subgroups, the CMS4 subtype, characterized by the upregulation of EMT-associated genes, unequivocally emerged as a distinct subtype with worse overall survival (OS) and relapse-free survival (RFS). Although CMS classification holds promise in future, at this time, its clinical application for risk-stratification in CRC patients remains unclear. Nonetheless, given the strong association of CMS4 subgroups with an EMT phenotype, there is an emerging interest to develop EMT-associated biomarkers, which may serve as surrogates for the CMS4 subtype, and may allow more improved patient stratification.

Recently, our group has shown that biomarkers highly expressed in liver metastasis are involved in distant metastasis and the EMT process [7, 8]. In this study, using a genomewide transcriptomic profiling of matched primary CRC and corresponding liver metastasis tissues, followed by their comparison in patients with and without disease recurrence, we identified a novel, EMT-related biomarker that robustly stratified low and high-risk CRC patients. Gene Set Enrichment Analysis (GSEA) revealed that high expression of integrin subunit beta like 1 (ITGBL1) strongly correlated with an EMT-phenotype, and significantly discriminated CRC patients with the CMS4 vs. the others subtypes. Subsequent clinical validation efforts revealed that high expression of *ITGBL1* associated with poor OS and RFS in multiple, large, independent CRC patient cohorts, which allowed us to conclude that ITGBL1 is an attractive and promising prognostic biomarker in CRC.

## Results and discussion

### Overexpression of metastatic-recurrence-related genes in CRC

**Fig. 1:**
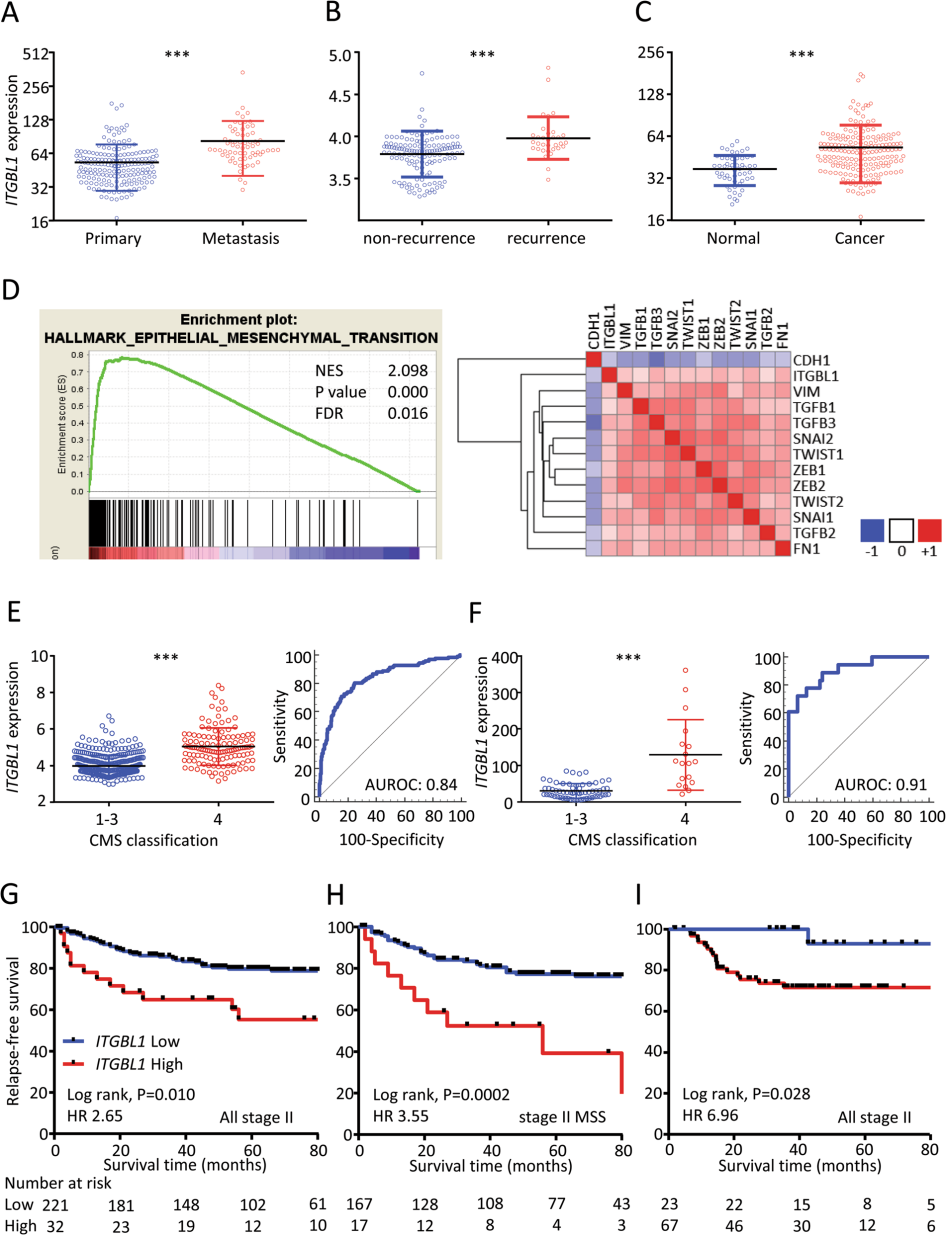
Biomarker discovery analysis in this study. *ITGBL1* expression was upregulated in various biomarker discovery analysis, **a**) Primary vs. metastasis tissues, **b**) patients with vs. without tumor recurrence, and **c**) normal vs. cancer tissues. **d** Enrichment plots of GSEA correlation analyses for *ITGBL1* with EMT-associated gene sets using the GSE39582 dataset (left). Heatmap for the correlation of *ITGBL1* and representative EMT-related genes by GENE-E software (right). *ITGBL1* expression is upregulated in the CMS4 subtype of CRCs in the two public datasets, **e**) GSE39582 dataset, and **f**) GSE33113 dataset. ***P < 0.001. Relationship between *ITGBL1* expression and RFS among patients **g**) in all stage II CRC patients with the GSE39582 cohort, **h**) in MSS stage II CRC patients within the GSE39582 cohort, and **i**) in all stage II CRCs in the GSE33113 cohort

We first used a systematic biomarker discovery step to identify metastatic recurrence-specific genes for CRC from the publicly available GSE17538 and GSE41258 datasets. We identified two genes, ITGBL1 and SPP1 (osteopontin), which were differentially expressed between the primary CRC vs. metastatic tissues, recurrence vs. non-recurrence groups and normal vs. cancers (> 2 fold change, and adjusted *P* < 0.05; Fig. 1a-c). Since, SPP1 has been extensively studied in CRC [9], while the clinical significance of ITGBL1 remains poorly but gaining a lot of attention in the field of cancer research [10], we selected ITGBL1 for further evaluation. The detailed methods are provided in the Additional file 1. The flow chart for the study design is illustrated in Additional file 2.

### *ITGBL1* expression strongly correlates with an epithelial mesenchymal transition in CRC

To gain further insight into the molecular function of ITGBL1 in CRC, we performed GSEA using genes that had a positive correlation with *ITGBL1* expression. Based on the normalized enrichment score (NES), the EMT gene set emerged to be most strongly correlated with *ITGBL1* expression (NES 2.099, *P* < 0.001, False discovery rate 0.016; Fig. 1d). Interestingly, several additional EMT-associated genes were also significantly correlated with the *ITGBL1* expression (Fig. 1d); suggesting that ITGBL1 expression may serve as an important indicator of an EMT phenotype in CRC. Recent evidence indicates that an EMT phenotype is associated with the dissociation of the primary tumor cells from the primary site, followed by intravasation into blood and/or lymphatic vessels, establishing metastasis [5]. Through such an EMT process, CRCs with High *ITGBL1* expression may lead to advanced disease, and present a higher risk for metastasis, which becomes the basis for developing recurrence prediction biomarkers.

### *ITGBL1* serves as a surrogate for predicting the CMS4 subtype in CRC

We next evaluated the expression of *ITGBL1* in the context of CMS status in two public datasets (GSE39582 and GSE33113). We found that *ITGBL1* expression was specifically higher in the CMS4 subtype vs. other subtypes in both patient cohorts. The AUROC for distinguishing CMS4 vs. CMS1–3 subtypes in CRC were 0.84 in GSE39582 and 0.91 in GSE33113 (Fig. 1e and f).

### *ITGBL1* expression associates with poor RFS in CRC patients

Furthermore, to investigate the clinical significance of *ITGBL1* expression for risk-stratification of disease recurrence in stage II CRC patients, the group in which adjuvant chemotherapy decision-making is most desirable, we analyzed RFS in patients from the GSE39582 and GSE33113 datasets (Fig. 1g and i, respectively). In line with our earlier findings, we observed that high *ITGBL1* expression group consistently demonstrated shorter RFS in stage II patients; yet again confirming the prognostic potential of this EMT-associated gene. In particular, based upon MSI analysis, high *ITGBL1* expression allowed identification of high-risk patients more effectively in microsatellite stable (MSS) stage II CRC patients vs. all stage II patients in the GSE39582 cohort (Fig. 1h**)**.

### The ITGBL1 protein expression is specifically higher in metastatic tissues from CRC patients

For a better understanding of the expression pattern of ITGBL1, we performed immunohistochemical (IHC) analysis. We found that ITGBL1 expression in normal colonic mucosa was quite weak (Additional file 3: Figure S2D). However, ITGBL1 expression gradually increased from the luminal region to the invasive front in primary CRC, indicating that elevation of ITGBL1 expression might facilitate higher metastatic potential at the invasive front in primary CRC (Additional file 3: Figure S2A, B, and C). Likewise, liver metastasis revealed extremely high expression of ITGBL1 compared to adjacent hepatocytes (Additional file 3: Figure S2E).

### High *ITGBL1* expression correlated with advanced stage, and presence of lymphovascular and distant metastasis in CRC patients

**Fig. 2:**
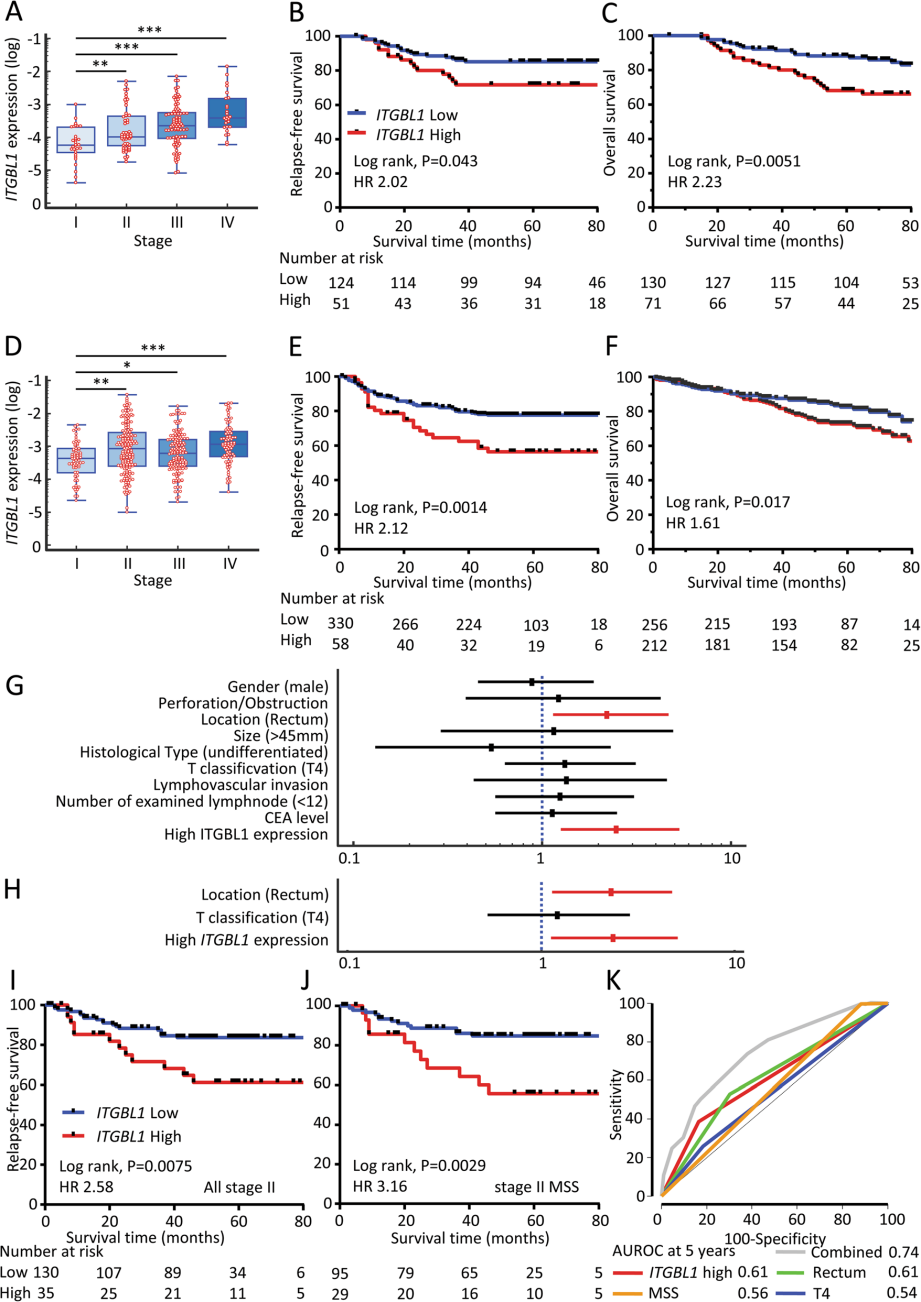
ITGBL1 expression in testing and validation clinical cohorts. Box plots representing *ITGBL1* levels in different Tumor Node Metastasis (TNM) stages (I, II, III, and IV) in CRC: **a**) The testing cohort (*N* = 201), and **d**) The validation cohort (*N* = 468). *P < 0.05; **P < 0.01; ***P < 0.001. The prognostic significance of *ITGBL1* expression was evaluated in CRC patients from two independent clinical cohorts: **b**, **c**) testing cohort, and **e**, **f**) validation cohort. Relapse-free survival in stage I-III patients (**b** and **e**) and overall survival in stage I-IV patients (**c** and **f**) were performed using the Kaplan–Meier test and the log-rank method. Forest plot of each clinicopathological factors, *ITGBL1* expression for predicting RFS in stage II CRC patients of validation cohort: **g**) Univariate analysis, and **h**) Multivariate analysis. Relationship between *ITGBL1* expression and RFS in stage II CRC patients of validation cohort: **i**) all stage II CRC patients, and **j**) MSS stage II CRC patients. **k**) Time-dependent ROC curves comparing and combining the predicting accuracy for recurrence at 5 years in stage II CRC patients

We next investigated the level of *ITGBL1* expression in relationship with various clinicopathological variables in two independent clinical testing and validation cohorts of 669 CRC patients (Additional file 4: Table S1). High *ITGBL1* expression significantly correlated with increased tumor size, higher T stage, lymphovascular invasion, and the presence of distant metastasis in both cohorts (Table 1). Furthermore, when all CRC patients were segregated based upon the TNM stage, a gradual increase in *ITGBL1* expression levels was observed from the low to high stages in both cohorts (Fig. 2a and d).

**Table 1:** Association between *ITGBL1* expression and clinicopathological factors

Variables	Testing cohort N (%)			Validation cohort N (%)		
	*ITGBL1* level			*ITGBL1* level		
	Low	High	*P* value	Low	High	*P* value
	N=130	N=71		N=256	N=212	
Gender						
Male	61	29	0.41	162	113	**0.03**
Female	69	42		94	99	
Age						
<65	80	45	0.88	114	81	0.19
≥65	50	26		142	131	
Location						
Colon	65	36	0.92	160	131	0.88
Rectum	65	35		96	81	
Histology						
Differentiated	119	68	0.39	239	191	0.23
Undifferentiated	11	3		17	21	
Tumor size (mm)						
≤45	81	29	**<0.01**	151	72	**<0.0001**
>45	49	42		91	137	
Unavailable	0	0		14	3	
T stage						
T1,T2	37	7	**<0.01**	68	24	**<0.0001**
T3,T4	93	64		188	188	
Lymphovascular invasion						
Absent	55	11	**<0.0001**	41	16	**<0.01**
Present	75	60		213	196	
Unavailable	0	0		2	0	
Lymph node Metastasis						
Absent	69	21	**0.001**	137	113	0.96
Present	61	50		119	99	
Distant metastasis						
Absent	120	57	**0.02**	224	163	**<0.01**
Present	10	14		32	49	
Stage						
I, II	67	18	**<0.001**	132	103	0.52
III, IV	63	53		124	109	
Preoperative CEA (ng/ml)						
<5	88	39	0.07	156	114	0.12
5≤	42	32		100	98

### Overexpression of *ITGBL1* correlated with poor survival in CRC patients

Next, we examined *ITGBL1* expression with regard to its prognostic significance in the testing (*n* = 201), and validation cohorts (*n* = 468). In both cohorts, we noted that high *ITGBL1* expression level correlated with shorter RFS in stage I-III patients (Fig. 2b and e), as well as a shorter OS in stage I-IV patients (Fig. 2c and f).

Cox’s univariate and multivariate analyses for RFS showed that high *ITGBL1* expression was an independent prognostic factor for RFS in stage II CRC patients in the validation cohort (Additional file 5; Fig. 2g and h); and was also found to be significant in predicting RFS with a HR of 2.58 (Fig. 2i). Specifically, as evidenced from the findings of the GSE39582 dataset, high *ITGBL1* expression could effectively identify high-risk patients in microsatellite stable (MSS) stage II CRC patients, whose risk stratification is very crucial for decision-making of the adjuvant therapy (HR 3.16; Fig. 2j). Taken together, these findings indicate that high *ITGBL1* expression has important clinical significance and could potentially serve as an important biomarker for predicting recurrence in CRC patients.

We finally constructed a RFS prediction model with various combinations of parameters including *ITGBL1* expression using the Cox’s proportional hazard model in stage II CRC patients. AUROC at five years of this prediction model including Rectum, T4, MSS and *ITGBL1* expression further improved from 0.61 to 0.74 (Fig. 2k); highlighting the recurrence predictive potential of *ITGBL1* in CRC.

## Conclusion

In conclusion, high *ITGBL1* expression in primary tumors was associated with recurrence in CRC patients following curative surgery. Our study identified ITGBL1 as a novel, promising EMT-associated gene that could help in risk stratification and recurrence prediction in CRC patients.

## Additional files

Additional file 1: Detailed materials and methods. (DOCX 40 kb)

Additional file 2: **Figure S1.** The study design. (DOCX 32 kb)

Additional file 3: **Figure S2.** IHC staining for ITGBL1. (DOCX 2489 kb)

Additional file 4: **Table S1.** The clinicopathological features of patients in this study. (DOCX 21 kb)

Additional file 5: **Table S2.** Univariate and multivariate analysis of RFS in stage II patients of validation cohort. (DOCX 23 kb)

## Abbreviations

AUROC: Area under the receiver operating curve
CMS: Consensus molecular subtypes
CRC: Colorectal cancer
EMT: Epithelial to mesenchymal transition
GSEA: Gene Set Enrichment Analysis
HR: Hazard ratio
ITGBL1: Integrin subunit beta like 1
MSS: Microsatellite stable
NES: Normalized enrichment score IHC Immunohistochemistry
OS: Overall survival
RFS: Relapse-free survival
