# Supplementary material for: Transcriptomic expression profiling identifies ITGBL1, an epithelial to mesenchymal transition (EMT)-associated gene, is a promising recurrence prediction biomarker in colorectal cancer
Source: Mol Cancer. 2019 Feb 4;18:19. doi: 10.1186/s12943-019-0945-y (PMC6360655; doi:10.1186/s12943-019-0945-y)
Supplement: Supplementary file 1 — Detailed materials and methods. (DOCX 40 kb) [file 12943_2019_945_MOESM1_ESM.docx]

**Materials and Methods**

Biomarker discovery analysis

To discover EMT and distant metastasis-associated markers for CRC recurrence after curative resection, we initially analyzed the GSE17538 dataset comprising of 173 stage I-III CRC patients (35 with recurrence and 138 without recurrence) and the GSE41258 dataset composed of 186 primary CRC, 67 metastatic CRC and 54 normal colonic mucosa samples [12, 13]. We selected candidate genes according to the following criteria: upregulated genes in the recurrence group vs. the non-recurrence group; upregulated genes in the metastatic vs. primary CRC; and upregulated genes in primary CRC vs. normal colonic mucosa. All comparisons were performed using GEO2R (fold change>2.0, adjusted P value<0.05) (Supplementary Figure 1).

To assess the prognostic significance of ITGBL1 and determine its biological mechanism in CRC, we also analyzed large public datasets from 656 colorectal cancer patients belonging to the GSE39582 [14] and GSE33113 datasets [15].

Patient and specimen collection

This study included examination a total of 669 primary fresh frozen CRC tissue specimens collected at the National Cancer Center Hospital from patients enrolled between 2004-2006 (testing cohort, N=201) and at the Tokyo Medical and Dental University Hospital between 2007-2011 (validation cohort, N=468). All specimens were stored at -80^o^C following treatment with *RNAlater* (QIAGEN, Hilden, Germany) until total RNA extraction was performed. Formalin-fixed paraffin-embedded (FFPE) tissues from stage II CRC patients in the validation cohort were used for microsatellite instability (MSI) analysis. There were no patients that received radiotherapy or chemotherapy before surgery in this study. All patients had sporadic CRC diagnosis and patients with hereditary cancer were not included in this study. We obtained written informed consent from all patients and received approval for this study from the institutional review boards of all participating institutions. For the OS and RFS, survival time was calculated from the date of surgery either to the each event or the last follow up date. OS and RFS events have been defined previously [9]. Details of the clinicopathological features of the patients involved in this study are in Table 1. A reporting recommendations for tumor marker prognostic studies (REMARK) [16] checklist is in Supplementary Table 1.

Total RNA extraction and cDNA synthesis

Fresh frozen specimens were homogenized with a Mixer Mill MM 300 homogenizer (QIAGEN). Total RNA from the fresh frozen tissues was extracted using the RNeasy Mini Kit (QIAGEN) according to the manufacturer’s instructions. Following that, cDNA was synthesized from 2 µg of total RNA using the High Capacity cDNA Reverse Transcription Kit according to the manufacturer’s instructions (Thermo Fisher Scientific, Waltham, MA). From FFPE specimens, genomic DNA was extracted using the Allprep FFPE kit (QIAGEN) according to the manufacturer’s instructions.

The Quantitative reverse transcription polymerase chain reaction (qRT-PCR)

The qRT-PCR assays were performed using the Fast SYBR Green Master Mix (Applied Biosystems, Foster City, CA). The relative expression of *ITGBL1* was normalized against ACTB using the 2-Δct method. The sequences of all primers used in this study are in the Supplementary Table 2.

Immunohistochemistry (IHC)

To determine the protein expression pattern of ITGBL1 in clinical tissue specimens, immunohistochemistry (IHC) was performed in 33 paired tissue samples of primary CRCs and adjacent normal mucosa, as well as 7 liver and lung metastases tissues. Four-µm-thick tissue sections were cut, de-paraffinized, subjected to antigen recovery treatment with 1 mM EDTA buffer target retrieval solution, and then autoclaved at 120°C for 10 minutes. Endogenous peroxidase activity was blocked by incubating with Dual Endogenous Enzyme Block (Dako, K4065, Carpinteria, CA) for 30 minutes. After washing with PBS, the sections were treated with 10% Normal Goat Serum blocking solution (Life Technologies, 50062Z, Carlsbad, CA). They were then incubated overnight at 4°C with an anti-ITGBL1 rabbit polyclonal antibody (Sigma-Aldrich, HPA005676, St. Louis, MO) diluted to 1:100. The subsequent staining was developed using the Dako Envision+Dual Link System horseradish peroxidase (HRP) method (Dako, K4065) and hematoxylin for nuclear counterstaining.

Gene set enrichment analysis of public microarray data

The association between the expression of *ITGBL1* and biological processes was analyzed using GSEA (GSEA v2.2.2, http://software.broadinstitute.org/gsea/index.jsp). Pearson correlations of *ITGBL1* expression (probe ID: 1557079_at) and expression of 54,675 probes within the GSE39582 dataset were calculated and genes were ranked accordingly. GSEA were conducted using this ranked gene list against hallmark gene sets from the Molecular Signatures Database v5.1 (Broad Institute, Cambridge, MA) [17, 18]. The default parameters of GSEA with gene lists of 15 to 500 genes were used, and analyses were run with 1,000 permutations. False discovery rate q-values <0.25 were considered statistically significant. We used GENE-E software (Broad Institute) for constructing heat maps and clustering to show the correlation of *ITGBL1* and representative EMT-related genes.

CMS classification

The colorectal cancer subtyping consortium provided the CMS status of two public databases (GSE39582 and GSE33113) [9].

Microsatellite instability analysis

MSI analysis was conducted using five mononucleotide repeat microsatellite markers (BAT-25, BAT-26, NR-21, NR-24, and NR-27) in a pentaplex PCR system. Primer sequences and MSI calling were described previously [19].

Statistical analysis

All statistical analysis was performed using the GraphPad Prism Ver. 6.0 (GraphPad Software, San Diego, CA) or Medcalc version 16.1 programs (MedCalc Software, Ostend, Belgium). Statistical differences between *ITGBL1* expression and various clinicopathological factors were determined by Wilcoxon’s signed rank test, the χ2 test, Fischer’s exact test or Mann-Whitney U test. Kaplan-Meier analysis and log-rank test were used to estimate and compare over-all survival and relapse-free survival rates of CRC patients with high and low *ITGBL1* expression. We dichotomized the *ITGBL1* expression values into high-expression and low-expression groups based on receiver operating characteristic (ROC) curves along with Youden’s index correction. The Cox’s proportional hazards models were used to identify independent prognostic factors dictating patient survival. We performed the time-dependent ROC analysis for investigating the prognostic accuracy. We used R software version 3.3.1 and the “survival ROC” package to analyze the time-dependent ROC curve. Correlation between two continuous values was analyzed by Spearman’s correlation. All P values were 2-sided, and those less than 0.05 were considered statistically significant.
