# Supplementary material for: Transcriptomic expression profiling identifies ITGBL1, an epithelial to mesenchymal transition (EMT)-associated gene, is a promising recurrence prediction biomarker in colorectal cancer
Source: Mol Cancer. 2019 Feb 4;18:19. doi: 10.1186/s12943-019-0945-y (PMC6360655; doi:10.1186/s12943-019-0945-y)
Supplement: Supplementary file 2 — Figure S1. The study design. (DOCX 32 kb) [file 12943_2019_945_MOESM2_ESM.docx]

**Supplementary Figure S1:** The Study Design

**Cohort 1 (Testing cohort)**

qRT-PCR(NCCH cohort)

N=201 (stages I-IV )

**Cohort 2 (Validation cohort)**

qRT-PCR(TMDU cohort)

N=468 (stages I-IV )

**Novel candidate biomarker for CRC**

ITGBL1

Excluded 1 well-studied gene

**Bioinformatics search for metastasis-related biomarkers for recurrence in CRC**

GSE41258 186 metastases vs. 67 primary CRC

GSE41258 67 primary CRC vs. 54 normal colon

GSE17538 35 recurrence vs. 138 non-recurrence

Identification of 2 genes
