# Supplementary figures and images for: Transcriptomic expression profiling identifies ITGBL1, an epithelial to mesenchymal transition (EMT)-associated gene, is a promising recurrence prediction biomarker in colorectal cancer
Source: Mol Cancer. 2019 Feb 4;18:19. doi: 10.1186/s12943-019-0945-y (PMC6360655; doi:10.1186/s12943-019-0945-y)

**
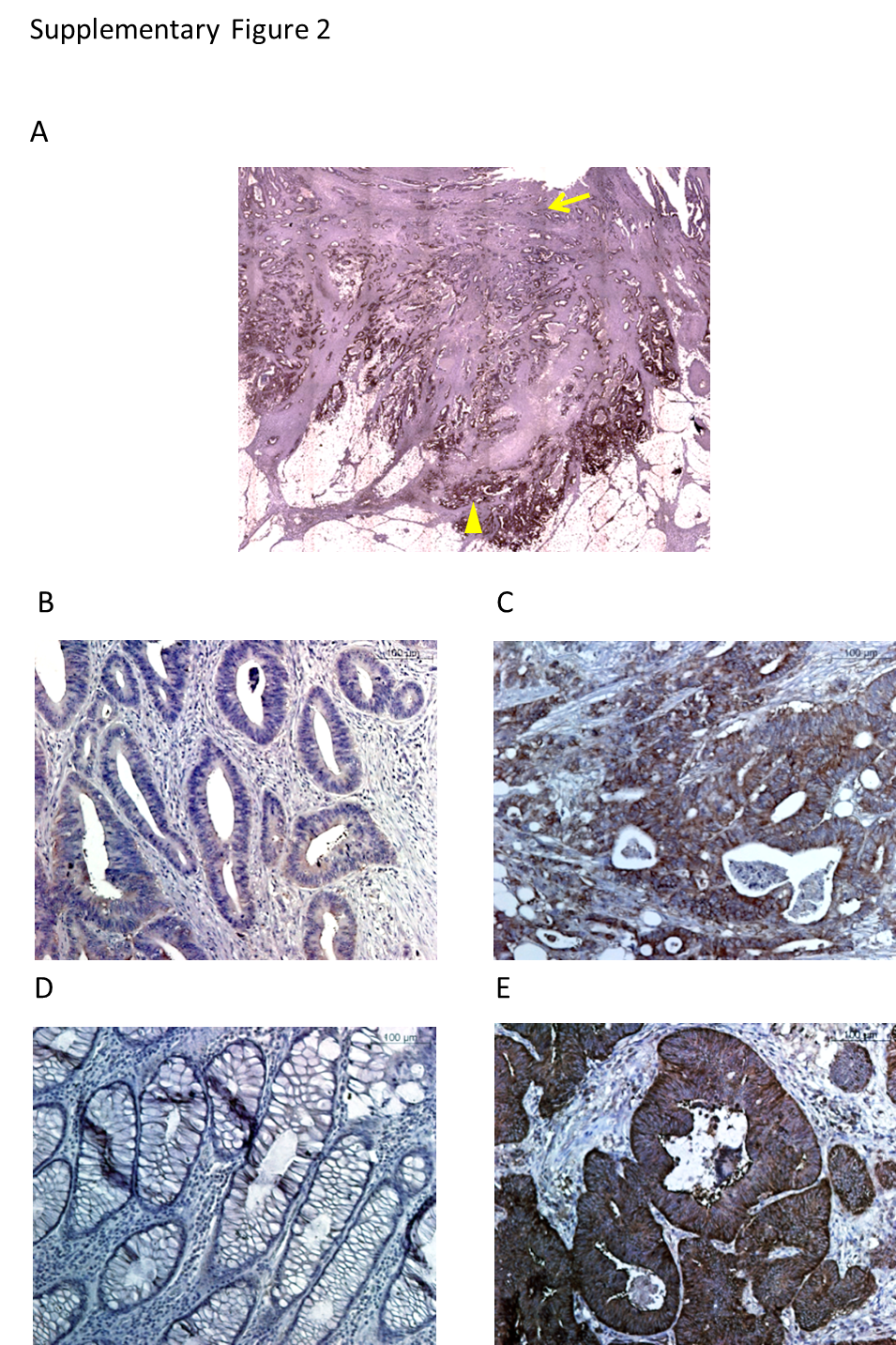
**

**Supplementary Figure S2:** The IHC staining for ITGBL1

Supplement: Supplementary file 3 — Figure S2. IHC staining for ITGBL1. (DOCX 2489 kb) [file 12943_2019_945_MOESM3_ESM.docx]
