# Supplementary material for: Transcriptomic expression profiling identifies ITGBL1, an epithelial to mesenchymal transition (EMT)-associated gene, is a promising recurrence prediction biomarker in colorectal cancer
Source: Mol Cancer. 2019 Feb 4;18:19. doi: 10.1186/s12943-019-0945-y (PMC6360655; doi:10.1186/s12943-019-0945-y)
Supplement: Supplementary file 4 — Table S1. The clinicopathological features of patients in this study. (DOCX 21 kb) [file 12943_2019_945_MOESM4_ESM.docx]

| **Table S1:** Clinicopathological features of patients in this study | | | |
| --- | --- | --- | --- |
|  |  | Testing Cohort N (%) | Validation cohort N (%) |
| Characteristics | | N=201 | N=468 |
| Gender |  |  |  |
|  | Male | 111 (55) | 275 (59) |
|  | Female | 90 (45) | 193 (41) |
| Age (Years) | |  |  |
|  | < 65 | 125 (62) | 195 (42) |
|  | ≥ 65 | 76 (38) | 273 (58) |
| Tumor Location | |  |  |
|  | Right sided colon | 35 (17) | 149(32) |
|  | Left sided colon | 66 (33) | 142 (30) |
|  | Rectum | 100 (50) | 177 (38) |
| Tumor Depth | |  |  |
|  | T1 | 4 (2) | 23 (5) |
|  | T2 | 40 (20) | 69 (15) |
|  | T3 | 104 (52) | 252 (54) |
|  | T4 | 53 (26) | 124 (27) |
| Histology (Differentiation) | |  |  |
|  | Differentiated | 187 (93) | 430 (91) |
|  | Undifferentiated | 14 (7) | 38 (9) |
| Lymphovascular Invasion | |  |  |
|  | Negative | 76 (38) | 57 (12) |
|  | Positive | 125 (62) | 409 (87) |
|  | Unavailable |  | 2 (1) |
| Lymph Node Metastasis | |  |  |
|  | Negative | 90 (45) | 250 (54) |
|  | Positive | 111 (55) | 218 (46) |
| Tumor Stage | |  |  |
|  | I | 30 (15) | 70 (15) |
|  | II | 55 (27) | 165 (35) |
|  | III | 90 (45) | 152 (32) |
|  | IV | 26 (13) | 81 (17) |
| Preoperative Serum CEA (ng/mL) | | |  |
|  | < 5 | 137 (68) | 271 (58) |
|  | ≥ 5 | 64 (32) | 197 (42) |
| Median follow up period (Months) | | |  |
|  |  | 68 | 52 |
