# Supplementary material for: Transcriptomic expression profiling identifies ITGBL1, an epithelial to mesenchymal transition (EMT)-associated gene, is a promising recurrence prediction biomarker in colorectal cancer
Source: Mol Cancer. 2019 Feb 4;18:19. doi: 10.1186/s12943-019-0945-y (PMC6360655; doi:10.1186/s12943-019-0945-y)
Supplement: Supplementary file 5 — Table S2. Univariate and multivariate analysis of RFS in stage II patients of validation cohort. (DOCX 23 kb) [file 12943_2019_945_MOESM5_ESM.docx]

| **Table S2: Univariate and multivariate analysis of RFS in stage II patients of validation cohort** | | | | |  | |  | |  |
| --- | --- | --- | --- | --- | --- | --- | --- | --- | --- |
|  | Univariate | | |  | Multivariate | | | | |
| Clinicopathological Variables | HR | 95%CI | P Value |  | HR | 95%CI | | P Value | |
| Gender (male) | 0.93 | 0.46 to 1.89 | 0.85 |  |  |  | |  | |
| Location (Rectum) | 2.32 | 1.15 to 4.69 | **0.019** |  | 2.33 | 1.14 to 4.76 | | **0.021** | |
| Perforation/Obstruction | 1.29 | 0.39 to 4.22 | 0.67 |  |  |  | |  | |
| Size (>45mm) | 1.20 | 0.29 to 4.99 | 0.80 |  |  |  | |  | |
| Histological Type (undifferentiated) | 0.56 | 0.13 to 2.33 | 0.53 |  |  |  | |  | |
| T classification (T4) | 1.39 | 0.63 to 3.11 | 0.55 |  | 1.24 | 0.53 to 2.87 | | 0.62 | |
| Lymphovascular invasion (positive) | 1.41 | 0.43 to 4.60 | 0.57 |  |  |  | |  | |
| Number of examined lymphnode (<12) | 1.31 | 0.57 to 3.05 | 0.52 |  |  |  | |  | |
| preoperative CEA level (≥ 5) | 1.19 | 0.57 to 2.47 | 0.65 |  |  |  | |  | |
| High *ITGBL1* expression | 2.58 | 1.26 to 5.30 | **0.010** |  | 2.40 | 1.13 to 5.08 | | **0.023** | |
